# Association between three-territory sign and prognosis of acute ischemic stroke patients with malignancy

**DOI:** 10.3389/fneur.2023.1265715

**Published:** 2023-09-28

**Authors:** Yuying Cheng, Yuye Ning, Yixin Zhao, Xiangqi Cao, Hui Liu, Tao Shi

**Affiliations:** ^1^Department of Cardiovascular Surgery, The First Affiliated Hospital of Xi’an Jiaotong University, Xi’an, China; ^2^Stroke Centre and Department of Neurology, The First Affiliated Hospital of Xi’an Jiaotong University, Xi’an, China; ^3^Innovation Center for Neurological Disorders and Department of Neurology, Xuanwu Hospital, Capital Medical University, Beijing, China; ^4^Biobank, The First Affiliated Hospital of Xi’an Jiaotong University, Xi’an, China

**Keywords:** three-territory sign, ischemic stroke, malignancy, atrial fibrillation, prognosis

## Abstract

**Background:**

Multiple cerebral infarcts are usually secondary to cardiogenic embolism, particularly through atrial fibrillation (AF). The three-territory sign (TTS) is an imaging marker that reflects multiple cerebral lesions involving three vascular territories measured by diffusion-weighted imaging (DWI), and the most common etiology is an underlying malignancy. Recent studies have shown that TTS is six times more frequently observed in acute ischemic stroke (AIS) patients with malignancy than in those with AF-related AIS. However, the relevance of TTS to the prognosis of IS patients with malignancy remains unclear.

**Methods:**

Over a 5-year period (May 2016 to 31 June 2021), AIS admissions with DWI were identified from the First Affiliated Hospital of Xi’an Jiaotong University. Patients were divided into two groups according to whether they had malignancy or AF, resulting in a total of 80 patients with known malignancy (malignancy group) and 92 patients with AF (AF group). All DWI images were reviewed to determine the territory lesion count. Demographic, clinical, and laboratory data, together with radiographic examination data and modified Rankin Scale (mRS) score within a year, were collected. The main outcome was the association between TTS and the prognosis of AIS patients with malignancy, analyzed by a multivariate logistic regression model.

**Results:**

A total of 172 patients met the selection criteria, including 17 (21.3%) patients in the malignancy group and 8 (8.7%) patients in the AF group with TTS. Age and sex distributions were similar for AIS patients of malignancy and AF. The TTS was 2.4 times more likely to be observed in AIS patients with malignancy compared to AF-related IS patients. The univariate analysis showed that hypertension (OR = 1.137, 95%CI: 1.002–1.291), D-dimer (OR = 1.328, 95%CI: 1.022–1.726), fibrin degradation product (OR = 1.117, 95%CI: 1.010–1.236), and lactate dehydrogenase (LDH; OR = 1.007, 95%CI: 1.000–1.015) were the risk factors for the high mortality rate. Multivariate analysis showed that TTS was the independent risk factor for mortality in AIS patients with malignancy (adjusted OR: 6.866, 95% CI: 1.371–34.395).

**Conclusion:**

TTS was more frequently observed in AIS patients with malignancy than AF-related AIS and substantially related to high poor outcome (mRS > 2) in AIS patients with malignancy, indicating diagnostic and prognostic value in malignancy-associated hypercoagulation stroke.

## 1. Introduction

Multiple acute ischemic stroke (MAIS) is most frequently attributed to an “embolic phenomenon,” such as atrial fibrillation (AF), the most common cause of cardioembolic stroke (1). MAIS also can be an initial presentation of malignancy that is commonly undiagnosed (2). Nearly 15% of patients with malignancy experience cerebrovascular events (3), and 1 in 10 patients admitted with acute ischemic stroke (AIS) have comorbid malignancy (4). Among the patients with comorbid AIS and malignancy, 25% die within 30 days, and the median survival is 4.5 months (5). Therefore, early identification of MAIS patients with malignancy is very important as patients with malignancy are often at increased risk of recurrent stroke, disability, and death (6).

Magnetic resonance imaging (MRI) using diffusion-weighted imaging facilitates the identification of ischemic infarction in malignancy as small infarctions that involve multiple vascular territories. Moreover, the number of territories involved is associated with the risk of disease (7, 8). Recent studies have shown that the three-territory sign (TTS), signifying three cerebral territory infarcts measured by DWI, is a highly specific marker of malignancy-associated AIS if there is no identifiable source of embolism (9). The TTS is six times more frequent in malignancy-related AIS than in AF-related AIS (10). However, studies evaluating imaging risk factors in patients with combined malignancy are lacking. Whether there is an association between TTS and the prognosis of AIS patients with malignancy has not been investigated.

Therefore, this study aimed to assess the clinical and imaging features of AIS patients with malignancy, investigate the incidence of TTS in AIS patients with malignancy compared to those with AF-related AIS, and explore the association between TTS and the prognoses of AIS patients with malignancy.

## 2. Materials and methods

### 2.1. Study population

This retrospective study collected anonymized clinical data of consecutively admitted patients with a primary diagnosis of all AIS from the Biobank of The First Affiliated Hospital of Xi’an Jiaotong University from 1 May 2016 through 31 June 2021. AIS patients (aged ≥18 years) meeting the following criteria were included: (1) diagnosis meeting the AIS criteria of the 2018 AHA/ACA guidelines (11); (2) having clear imaging evidence on DWI and 24-h electrocardiography (ECG) recording; (3) within 7 days of onset. We excluded patients who had the following characteristics: (1) ≥50% luminal stenosis in extracranial or intracranial arteries; (2) multiple etiologies; (3) poor prognosis as a result of tumor progression or treatment. Based on the presence or absence of malignancy or AF, participants were assigned to two groups (malignancy group and AF group). A chart review for all patients was completed, evaluating clinical data, medical history, demographics, and imaging. This research was approved by the Ethics Committee of the First Affiliated Hospital of Xi’an Jiaotong University (No. XJTU1AF2021LSK-117).

### 2.2. Clinical variables and outcome measurements

The baseline demographic information of the patients was abstracted from the electronic medical records, including sex, age, risk factors for atherosclerosis, type of malignancy, and laboratory data. The included patients had adequate clinical evaluations, which were as follows: vital sign monitoring, brain/chest CT and brain MRI, ultra-sound of cervical vessels, 24-h ECG, echocardiography, intravenous ultrasound, and laboratory tests (routine hematology and biochemistry). The modified Rankin scale (mRS) within a year of follow-up was determined by the patients’ medical records from outpatient clinics or by telephone. The primary outcome was the association between TTS and prognosis of AIS patients with malignancy, and the second outcome was the incidence of TTS in AIS patients with malignancy.

### 2.3. Imaging assessment

All participants were scanned using the same 3.0-T Siemens Skyra scanner (Siemens AG, Munich, Germany) with a completed neuroradiology report. We analyzed the DWI imaging features of all participants and recorded the vascular territories involved. For imaging, three ischemic stroke MRI-DWI patterns were identified in both groups as follows (9, 10): One Territory: involving the anterior or the posterior circulation; Two Territory: involving the anterior circulation plus posterior circulation or bi-lateral anterior circulation; Three Territory: involving the bilateral anterior and posterior circulation.

### 2.4. Statistical analysis

The data are expressed as mean ± standard deviation (SD) for continuous variables and as percentages for categorical variables. We performed statistical assessments between the groups using t-test, χ^2^, or Fisher tests to establish differences between the clinical groups. One-way analysis of variance was used to make the mean comparisons between the groups. To examine the correlation between TTS and prognosis, a binary logistic regression model was used to calculate the adjusted odds ratio (OR) and 95% confidence intervals (CIs). Multivariate logistic regression analysis was also applied to identify independent risk factors for prognosis. A *p* < 0.05 was considered statistically significant. All statistical analysis was performed by the SPSS version 26.0 (Chicago, IL, USA).

## 3. Results

### 3.1. Clinical and imaging characteristics

In the study, 9,160 patients were diagnosed with IS, and 550 patients were enrolled according to the inclusion and exclusion criteria. According to the TOAST classification criteria (12), 92 patients were diagnosed with AF-related AIS without malignancy, and 131 patients had a history of malignancy before admission. Among the 131 patients with malignancy, 51 were excluded because they had AF. A total of 172 individuals were included in the analyses (mean ± standard deviation age = 72.57 ± 10.63, 51.2% male). This yielded 80 patients (age 73.36 ± 10.32 years, 53.8% male) in the malignancy group (malignancy and no AF) and 92 patients (age 71.88 ± 10.89 years, 48.9% male) in the AF group (AF and no malignancy).

**Figure 1:**
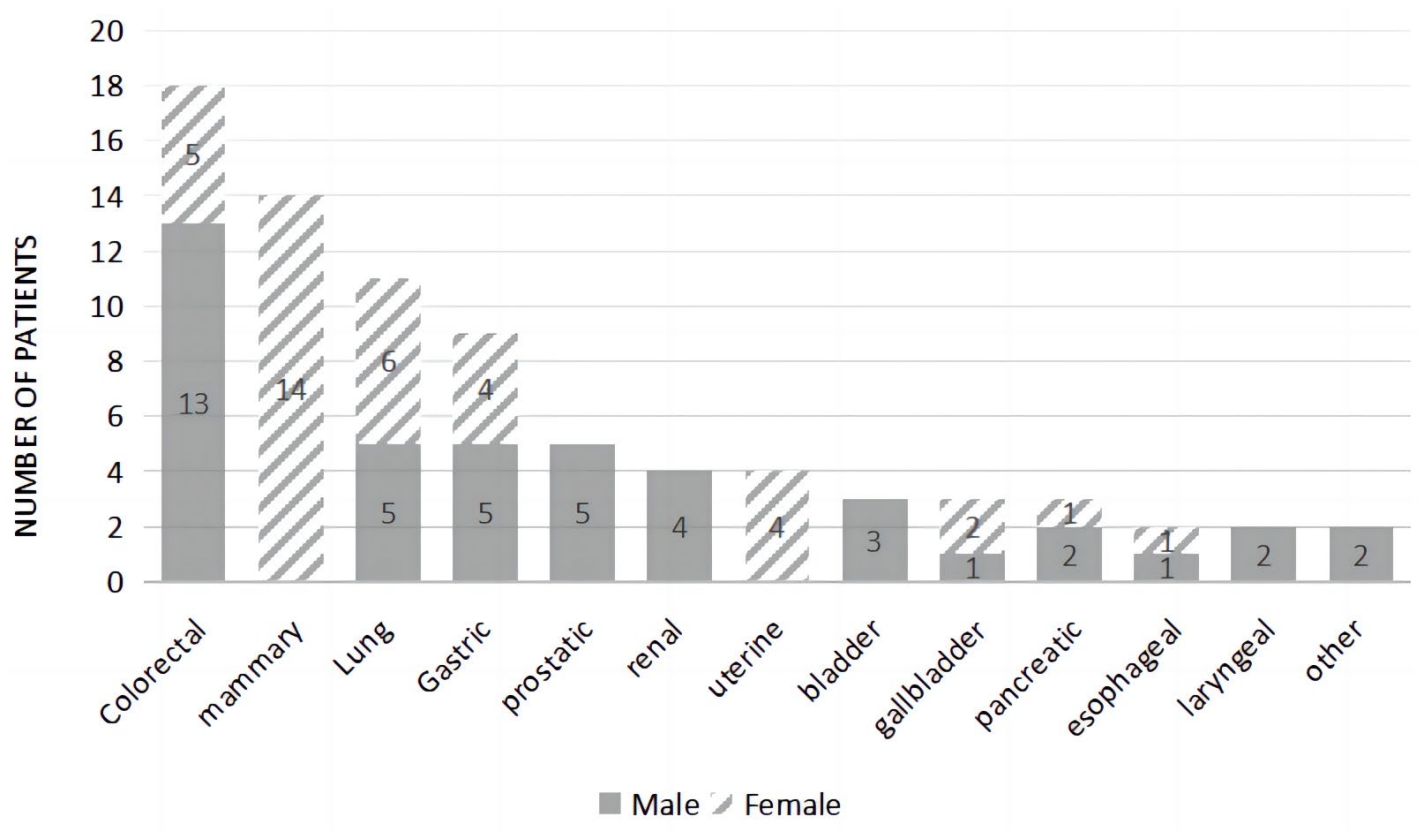
Primary malignancy location and sex ratio.

The three most common malignancies were colorectal, mammary, and lung in origin (Figure 1). The malignancy types for this group were as follows: colorectal cancer (22.50%), mammary cancer (17.50%), lung cancer (13.75%), gastric cancer (11.25%), prostate cancer (6.25%), renal cancer (5.00%), uterine cancer (5.00%), bladder cancer (3.75%), gallbladder cancer (3.75%), pancreatic cancer (3.75%), esophageal cancer (2.50%), laryngeal cancer (2.50%), liver cancer (1.25%), and skin cancer (1.25%). During the follow-up period of 1 year, 41.3% of patients died.

**Figure 2:**
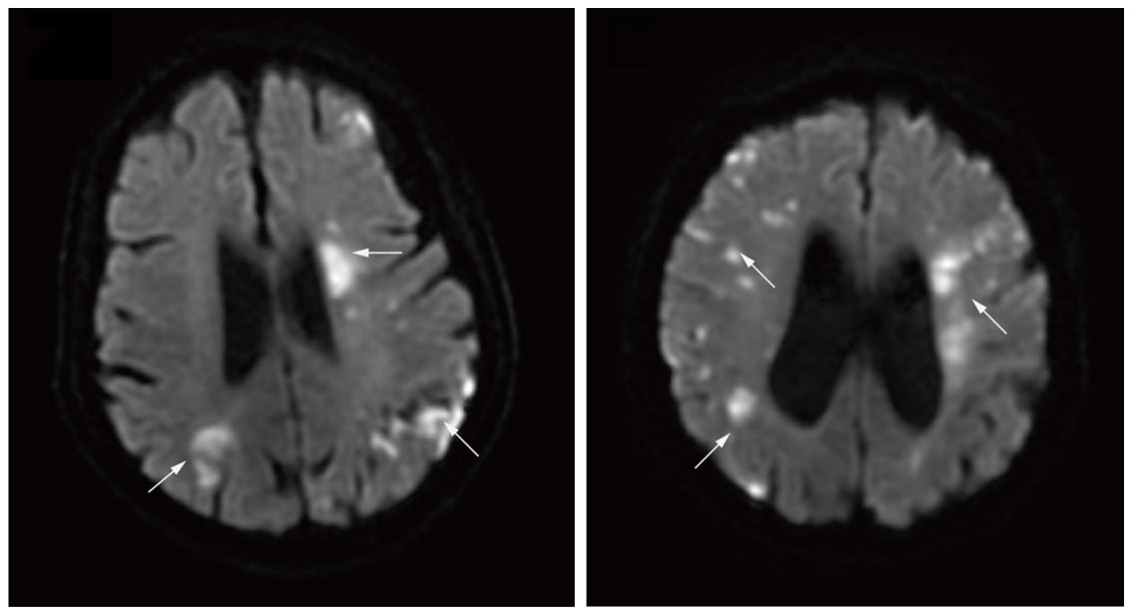
Diffusion-weighted magnetic resonance imaging demonstrating multiple high intensity areas (arrows) in all three major arterial territories of the brain.

Age and sex distributions were similar for the malignancy group and the AF group (Table 1). Patients in the malignancy group had a lower incidence of coronary heart disease (CHD) and hyperhomocysteinemia (*p* < 0.05). Low-density lipoprotein (LDL) and eosinophils values were higher in the malignancy group than in the AF group, while red blood cell (RBC) and hemoglobin (Hb) counts were lower (*p* < 0.05). In the malignancy group, MRI assessment showed multiple scattered lesions. Patients with two- and three-territory signs accounted for 49 (28.5%) and 25 (14.5%), respectively. Figure 2 shows brain MRI scans of two AIS patients with malignancy, revealing multiple high-intensity areas.

**Table 1:** Clinical and imaging characteristics of AIS patients in the malignancy group and AF group.

	Total (*n* = 172)	Malignancy group (*n* = 80)	AF group (*n* = 92)	*p*-value
Male, *n* (%)	88 (51.2)	43 (53.8)	45 (48.9)	0.544
Age, years	72.57 ± 10.63	73.36 ± 10.32	71.88 ± 10.89	0.363
Hypertension, *n* (%)	102 (59.3)	50 (62.5)	52 (56.5)	0.441
Diabetes, *n* (%)	45 (26.2)	19 (23.8)	26 (18.3)	0.602
Hyperlipidemia, *n* (%)	28 (16.3)	17 (21.3)	11 (12.0)	0.146
CHD, *n* (%)	47 (27.3)	15 (18.8)	32 (34.8)	0.025
Anemia, *n* (%)	50 (29.1)	33 (41.3)	17 (18.5)	0.001
RBC (×10^12^/L)	4.04 ± 0.68	3.87 ± 0.69	4.20 ± 0.63	0.002
WBC (×10^9^/L)	7.84 ± 3.86	7.41 ± 4.30	8.21 ± 3.41	0.187
PLT (×10^9^/L)	200.56 ± 91.18	195.58 ± 100.41	205.01 ± 82.40	0.512
Hb, g/L	122.57 ± 23.13	114.86 ± 23.75	129.48 ± 20.33	0.001
TC, μmol/L	3.91 ± 0.98	4.04 ± 1.02	3.78 ± 0.93	0.105
LDL, mmol/L	2.16 ± 0.80	2.32 ± 0.81	2.02 ± 0.78	0.026
HDL, mmol/L	1.04 ± 0.26	1.00 ± 0.26	1.07 ± 0.26	0.128
Hcy, μmol/L	20.14 ± 13.52	17.68 ± 9.88	22.30 ± 15.78	0.038
D-dimer, mg/L	4.30 ± 9.65	5.55 ± 10.26	3.30 ± 9.05	0.152
Fib, g/L	3.79 ± 1.40	3.73 ± 1.28	3.85 ± 1.50	0.594
FDP, μg/ml	11.40 ± 24.60	15.54 ± 28.61	7.86 ± 20.01	0.052
NE (×10^9^/L)	5.96 ± 3.61	5.61 ± 4.14	6.27 ± 3.05	0.242
EOS (×10^9^/L)	0.13 ± 0.12	0.15 ± 0.13	0.11 ± 0.11	0.036
LDH, U/L	317.56 ± 471.55	283.66 ± 178.45	341.20 ± 596.69	0.498
ALB, g/L	36.27 ± 5.12	35.92 ± 5.26	36.55 ± 5.03	0.464
One-territory sign	98 (57.0)	40 (50.0)	58 (63.0)	0.092
Two-territory sign	49 (28.5)	23 (28.7)	26 (28.3)	1.000
Three-territory sign	25 (14.5)	17 (21.3)	8 (8.7)	0.029
Mortality	71 (41.3)	48 (60.0)	23 (25.0)	0.000
mRS > 2	124 (72.1)	73 (78.8)	61 (66.3)	0.088

Values are means (SD) or medians (IQR) or numbers (%). AIS, acute ischemic stroke; AF, atrial fibrillation; CHD, coronary heart disease; RBC, Red blood cell; WBC, White blood cell; PLT, Platelet; Hb, Hemoglobin; TC, total cholesterol; TG, triglycerides; LDL, low-density lipoprotein; HDL, high-density lipoprotein; Hcy, homocysteine; Fib, fibrinogen; FDP, fibrinogen degradation product; NE, neutrophils; EOS, eosinophils; LDH, lactate dehydrogenase; ALB, albumin; mRS, the modified Rankin scale.

### 3.2. Association between clinical features and prognostic factors

**Figure 3:**
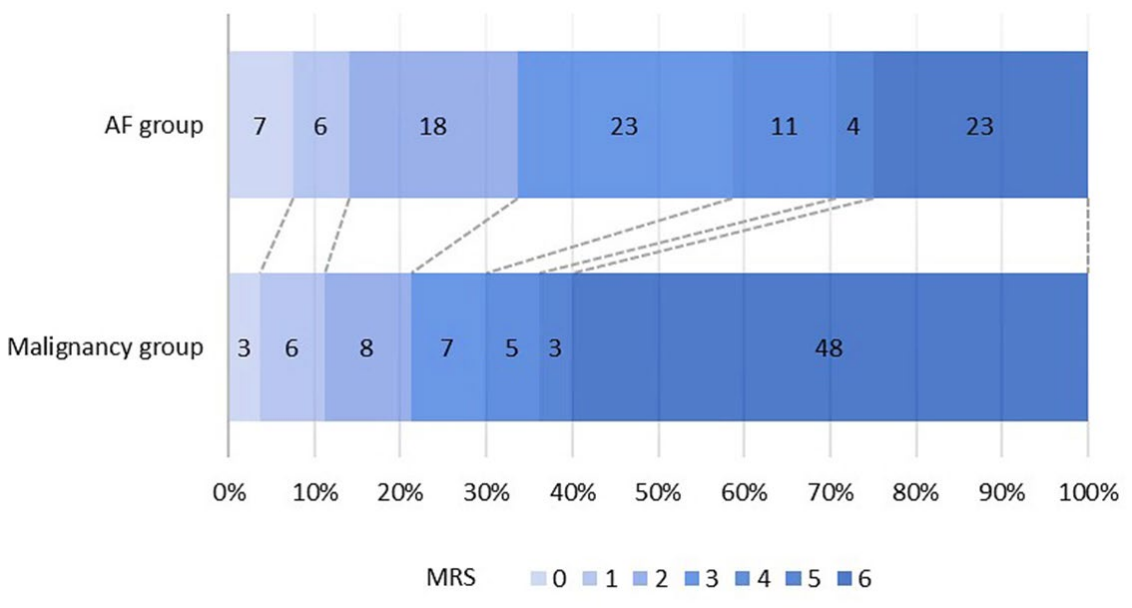
Distribution of 6 months modified Rankin scale (mRS).

The distribution of mRS scores of the two groups is shown in Figure 3. A poor outcome (mRS score of 3–6) was found in 63 (78.8%) patients in the malignancy group and 61 (66.3%) patients in the AF group, compared to an mRS of 0–2 before stroke onset. A total of 48 (60.0%) patients died in the malignancy group and 23 (25.0%) in the AF group. Table 1 shows that the difference in mortality between the two groups was statistically significant (*p* < 0.001).

Of the patients within the malignancy group, 40 (50.0%) patients had a 1-territory infarct pattern, and 23 (28.7%) patients had a 2-territory pattern. The TTS was observed in 17 patients (21.3%), representing one-fifth of patients with malignancy. In the AF group (*n* = 92), 58 (63.0%) patients had a 1-territory stroke pattern, 26 (28.3%) had a 2-territory pattern, and only 8 (8.7%) had the TTS. The overall association between the number of territories and the diagnostic group was significant, and there was also a significant difference in the number of patients with the TTS (*p* = 0.029). Furthermore, the TTS was 2.4 times more likely to be present in the malignancy group than in the AF group.

There were significant associations between clinical features and prognostic factors in AIS patients with malignancy (Table 2). In the univariate analysis, hypertension, D-dimer, and fibrin degradation product (FDP) were associated with mortality. Anemia, white blood cell (WBC) count, blood platelet (PLT) level, hemoglobin (Hb) count, D-dimer, FDP, neutrophil count, and albumin level were all found to be associated with TTS.

**Table 2:** Association between clinical features and prognostic factors in AIS patients with malignancy.

	Functional outcome (mRS > 2)			Mortality			Three-territory signs		
	*p*-value	OR	95%CI	*p*-value	OR	95%CI	*p*-value	OR	95%CI
Male, *n* (%)	0.637	0.770	0.260–2.279	0.411	0.684	0.277–1.690	0.534	0.711	0.243–2.082
Age, years	0.258	0.968	0.916–1.024	0.543	0.986	0.944–1.031	0.110	0.958	0.909–1.010
Hypertension, *n* (%)	0.440	0.633	0.199–2.018	0.021	0.304	0.111–0.837	0.361	0.604	0.204–1.785
Diabetes, *n* (%)	0.981	1.016	0.288–3.587	0.202	0.508	0.179–1.438	0.538	1.458	0.439–4.844
CHD, *n* (%)	0.896	1.098	0.272–4.437	0.560	0.714	0.231–2.213	0.571	1.455	0.398–5.315
Hyperlipidemia, *n* (%)	0.358	0.565	0.167–1.909	0.224	0.511	0.173–1.508	0.796	1.183	0.330–4.239
Anemia, *n* (%)	0.575	1.375	0.452–4.184	0.578	1.296	0.519–3.237	0.008	4.800	1.494–15.425
RBC (×10^12^/L)	0.079	0.460	0.193–1.096	0.122	0.579	0.290–1.157	0.023	0.371	0.158–0.874
WBC (×10^9^/L)	0.486	1.060	0.900–1.248	0.065	1.177	0.990–1.400	0.017	1.205	1.034–1.405
PLT (×10^9^/L)	0.837	1.001	0.995–1.006	0.935	1.000	0.995–1.004	0.028	0.990	0.981–0.999
Hb, g/L	0.558	0.993	0.970–1.017	0.235	0.988	0.969–1.008	0.003	0.958	0.932–0.985
TC, μmol/L	0.909	1.032	0.604–1.761	0.757	1.074	0.684–1.687	0.859	0.951	0.459–1.647
LDL, mmol/L	0.378	0.736	0.372–1.456	0.698	0.888	0.489–1.615	0.827	0.918	0.427–1.977
HDL, mmol/L	0.447	2.425	0.248–23.757	0.733	0.721	0.110–4.738	0.474	0.406	0.034–4.798
Hcy, μmol/L	0.669	0.988	0.936–1.043	0.343	0.975	0.926–1.027	0.584	0.980	0.910–1.055
D-dimer, mg/L	0.208	1.132	0.933–1.372	0.034	1.328	1.022–1.726	0.014	1.080	1.016–1.148
Fib, g/L	0.948	0.985	0.631–1.538	0.754	0.943	0.652–1.364	0.079	0.637	0.385–1.053
FDP, μg/ml	0.164	1.049	0.981–1.122	0.031	1.117	1.010–1.236	0.007	1.032	1.008–1.056
NE (×10^9^/L)	0.450	1.071	0.896–1.280	0.060	1.201	0.992–1.452	0.010	1.266	1.057–1.515
EOS (×10^9^/L)	0.276	0.123	0.003–5.323	0.097	0.039	0.001–1.806	0.062	0.002	0.000–1.392
LDH, U/L	0.174	1.005	0.998–1.011	0.037	1.007	1.000–1.015	0.003	1.006	1.002–1.010
ALB, g/L	0.093	0.897	0.789–1.018	0.064	0.904	0.813–1.006	0.011	0.843	0.739–0.962

Values are means (SD) or medians (IQR) or numbers (%). OR, Odds ratio; 95% CI, 95% confidence interval; CHD, coronary heart disease; RBC, Red blood cell; WBC, White blood cell; PLT, Platelet; Hb, Hemoglobin; LDL, low-density lipoprotein; HDL, high-density lipoprotein; Hcy, homocysteine; Fib, fibrinogen; FDP, fibrinogen degradation product; NE, neutrophils; EOS, eosinophils; LDH, lactate dehydrogenase; ALB, al-bumin; mRS, the modified Rankin scale.

### 3.3. Association between three-territory sign and prognosis

In the binary logistic regression model analysis, TTS was statistically significantly associated with mortality in AIS patients with malignancy (unadjusted OR: 6.818, 95% CI: 1.438–32.319, *p* = 0.016; Table 3). The effect of malignancy was significant in both models. Model 1, after adjusting for age and sex, showed an adjusted OR of 6.568 (95% CI: 1.316–31.701, *p* < 0.001), while Model 2 had an OR of 6.866 (95% CI, 1.371–34.395). The results of the multivariate analysis are shown in Table 3.

**Table 3:** Univariate and multivariate logistic regression for the outcomes by three-territory signs.

	Functional outcome (mRS > 2)			Mortality		
	*p*-value	OR	95%CI	*p*-value	OR	95%CI
Univariate analysis	0.113	5.447	0.668–44.409	0.016	6.818	1.438–32.319
Multivariate analysis						
Model 1	0.147	4.799	0.578–39.880	0.019	6.568	1.361–31.701
Model 2	0.15	4.743	0.570–39.477	0.019	6.866	1.371–34.395

Model 1 was adjusted for demographics, including age, and sex. Model 2 was adjusted for variables in model 1, plus history of hypertension. OR, Odds ratio; 95% CI, 95% confidence interval; mRS, the modified Rankin scale.

## 4. Discussion

The presence of the TTS in AIS patients with malignancy is still inadequately documented and there are no studies evaluating the association between the TTS and the prognosis in patients with malignancy (10). In this retrospective study, we discovered that images of patients with malignancy-related AIS are 2.4 times more likely to exhibit the TTS compared to those with AF-related AIS. Previous studies have shown that in AIS patients with malignancy, the incidence of infarction involving 3 vascular territories is 6 times higher than those with AF (10), while the present study found only a 2.4-fold increase in infarction. The inconsistency in these results may merely represent TTS being more prevalent in AIS patients with malignancy. Thus, it is reasonable to assess the potential for malignancy in AIS patients with a TTS of undetermined etiology. In addition, the TTS was strongly associated with increased mortality in AIS patients with malignancy, even after adjustment for age, sex, or other vascular risk factors. This result suggests that the TTS may be a clinically meaningful indicator of increased mortality in AIS patients with malignancy.

It has been reported that the risk of mortality associated with AIS was higher in patients with malignant tumors than those without such tumors (13). This is consistent with TTS being significantly associated with mortality (unadjusted OR: 5.785, 95% CI: 2.175–15.386, *p* < 0.001) in AIS patients with malignancy. Furthermore, studies have shown that acute infarction in ≥2 territories was observed in 84% of patients with malignancy who also had D-dimer levels >3 μg/ml (14). In the current study, 23 (28.75%) patients in the malignancy group and 23 (25.00%) patients in the AF group had D-dimer levels >3 μg/ml, demonstrating no statistical difference between groups. Most notably, the mortality rate in the present study (25.0%) was higher compared to that reported in previous studies. This difference can likely be attributed to variations in the age and disease severity among the patients included in the studies. The average age of AF-related AIS patients in the current study was 71.88 ± 10.89 years, with 22 (23.91%) being over 80 years old. As a specialized stroke center in the area, we admit patients with more severe AIS were referred from other hospitals, meaning that the majority of AIS patients received endovascular therapy, with some having an onset time of longer than 24 h. Furthermore, our findings did not suggest any association between the prognosis (mRS > 2) of malignancy-related AIS patients and previously reported clinical parameters including elevated D-dimer (15), CRP (16), and eosinophils (17), or underlying co-morbidities (18). In the current study, although there was no statistically significant difference in follow-up mRS between AIS patients with and without malignancy, there was a statistically significant difference in the mortality rate between the two groups (*p* < 0.001). Further studies involving larger patient numbers are needed to clarify this finding. An investigation for underlying malignancy is valuable in the presence of the TTS, especially if echocardiography and ambulatory ECG do not demonstrate valvular disease, intracardiac thrombus, or AF. Furthermore, the TTS may offer important diagnostic information to facilitate the identification of other under-recognized causes of stroke.

The limitations of our study include its small sample size and being a single-center retrospective study. Because the sample size was small, other characteristics of AIS patients with malignancy could not be adequately evaluated, which may lead to a failure to obtain accurate results. Furthermore, the clinical data available from the national biobank studies are incomplete, leading to an absence of many useful markers (constituting an information bias), such as cancer stage and treatment given.

## 5. Conclusion

In AIS patients with malignancy, imaging is characterized by the TTS, which is observed more frequently than in AF-related AIS. The TTS is the independent prognostic risk factor of poor outcome (mRS > 2) in AIS patients with malignancy. In patients with TTS of undetermined etiology, it is valuable to investigate the presence of an underlying malignancy.

## Data availability statement

The original contributions presented in the study are included in the article/supplementary material, further inquiries can be directed to the corresponding author.

## Ethics statement

The studies involving humans were approved by the First Affiliated Hospital of Xi'an Jiaotong University (No. XJTU1AF2021LSK-117). The studies were conducted in accordance with the local legislation and institutional requirements. The human samples used in this study were acquired from primarily isolates as part of our previous study for which ethical approval was obtained. Written informed consent for participation was not required from the participants or the participants' legal guardians/next of kin in accordance with the national legislation and institutional requirements.

## Author contributions

YC: Formal analysis, Writing – original draft, Writing – review & editing. YN: Formal analysis, Writing – original draft, Writing – review & editing. YZ: Data curation, Formal analysis, Methodology, Writing – original draft. XC: Data curation, Formal analysis, Investigation, Methodology, Writing – original draft. HL: Data curation, Methodology, Writing – review & editing. TS: Conceptualization, Investigation, Writing – original draft, Writing – review & editing.
